# Dynamic Changes in pStat3 Are Involved in Meiotic Spindle Assembly in Mouse Oocytes

**DOI:** 10.3390/ijms21041220

**Published:** 2020-02-12

**Authors:** Seiki Haraguchi, Mitsumi Ikeda, Satoshi Akagi, Yuji Hirao

**Affiliations:** 1 Animal Biotechnology Unit, Division of Animal Sciences, Institute of Agrobiological Sciences, NARO, 2 Ikenodai, Tsukuba, Ibaraki 305-0901, Japan; mtmikd@affrc.go.jp; 2 Embryo Production Research Unit, Division of Animal Breeding and Reproduction Research, Institute of Livestock and Grassland Science, NARO, 2 Ikenodai, Tsukuba, Ibaraki 305-0901, Japan; akagi@affrc.go.jp (S.A.); yujih@affrc.go.jp (Y.H.)

**Keywords:** Stat3, pStat3, oocyte maturation, meiosis, spindle assembly, MTOCs

## Abstract

The signal transducer and activator of transcription 3 of the pStat3 inhibitors, Stattic, and (Stat3) is activated upon phosphorylation at Y705 (pStat3) and serves the dual function of signal transduction and transcription activation. Our previous study suggested that pStat3 is functional during oocyte maturation when transcription is silenced. Therefore, we speculated that pStat3 serves other functions. Immunocytochemical analysis revealed that pStat3 emerges at microtubule asters and spindle and is subsequently localized at the spindle poles along with pericentrin during mouse oocyte maturation. Both Stat3 and pStat3 proteins were detected in conditionally knocked out *Stat3^−/^^−^* mouse oocytes. pStat3 localization was the same in *Stat3^+/^^+^* and *Stat3^−/^^−^*oocytes, and oocyte maturation proceeded normally, suggesting that pStat3 was still functional. Furthermore, the treatment of oocytes with the Stat3-specific inhibitors stattic and BP-1-102 or anti-pStat3 antibody led to significantly abnormal spindle assembly and chromosome mislocation in a dose-dependent manner, and pStat3 was either absent or improperly localized in these oocytes. Moreover, the development of pre-implantation stage embryos derived from inhibitor-treated oocytes was significantly hampered following in vitro fertilization. These findings indicate a novel function of pStat3 in spindle assembly.

## 1. Introduction

Many studies have demonstrated the dual function of signal transducer and activator of transcription 3 (Stat3) in signal transduction and transcription activation. Stat3 is involved in numerous biological processes, such as cell proliferation, differentiation, and survival. Although Stat3 is localized to the cytoplasm in its inactive form, stimulation by cytokines or growth factors triggers its phosphorylation (pStat3) at the tyrosine residue (Y705), inducing dimerization, nuclear translocation, and DNA binding [1,2,3]. Janus kinases are one of the best-studied upstream kinases involved in the Jak/Stat pathway [4]. Stat3 contains the Src homology-2 (SH2) domain required for the dimerization and activation of pStat3 monomers [5,6,7]. A transcription-independent mechanism of Stat3 has been reported through which cytoplasmic Stat3 modulates microtubule dynamics and cell migration via interaction with stathmin [8,9]. Moreover, Morris et al. [10] have reported that Stat3 plays a role in the regulation of centrosome clustering in cancer cells.

The Stat3 protein is expressed in the oocytes of mammals, including mouse [11,12,13], human [11], and pigs [14]. We previously reported that Stat3 is activated by leukemia inhibitory factor and promotes a part of oocyte maturation in pigs [14]. These findings suggest that pStat3 is functional during oocyte maturation. In mice, maternal mRNAs are expressed and accumulated during the growing phase [15,16]. Appropriate maternal transcriptome is achieved via proper uridylation and polyadenylation of tail length [17]. However, transcriptional activity is repressed in fully grown germinal vesicle (GV) oocytes [18,19]. Following stimulation with the preovulatory LH surge, meiosis resumes and the oocytes undergo germinal vesicle break down (GVBD). Until zygotic gene activation at the two-cell stage, oocytes rely on the maternal transcriptome [20]. Thus, transcriptional repression is vital for subsequent pre-implantation stage development [17,21].

*Stat3*-deficient mice exhibit embryonic lethality after implantation (E 7.0) [22]. Consequently, a conditional strategy such as the use of the Cre-loxP system is required to create genetically knocked out maternal *Stat3* (*Stat3^−/^^−^*) oocytes. Cre driver mice, such as *Zp3-Cre* or *Gdf9-Cre* transgenic (Tg) mice, are commonly used for the conditional expression of Cre recombinase in oocytes. In *Zp3-Cre* Tg mice, Cre expression is induced early in growing oocytes at the primary or the secondary follicular stages [23,24]. Oocytes with conditionally deleted Stat3 showed normal maturation, fertility, and pre-implantation development [25,26]. Thus, maternal Stat3 expressed before Cre likely remains functional in *Stat3^−/^^−^* oocytes.

Maturing mouse oocytes are thought to be an ideal model for studying the transcription-independent function of Stat3 as transcription is repressed during this stage. In this study, we first revealed pStat3 expression patterns in maturing mouse oocytes. Moreover, we examined the phenotype of pStat3 disruption in oocytes treated with Stat3-specific inhibitors and anti-pStat3 antibody in *Stat3^+/^^+^* and *Stat3^−/^^−^* oocytes. Here, we report that pStat3 is localized at the microtubule-organizing centers (MTOCs) and plays an important role in spindle assembly and chromosome segregation.

## 2. Results

### 2.1. Changes in Relative Stat3 and pStat3 Expression from Oocyte Maturation to Pre-Implantation Stages

**Figure 1:**
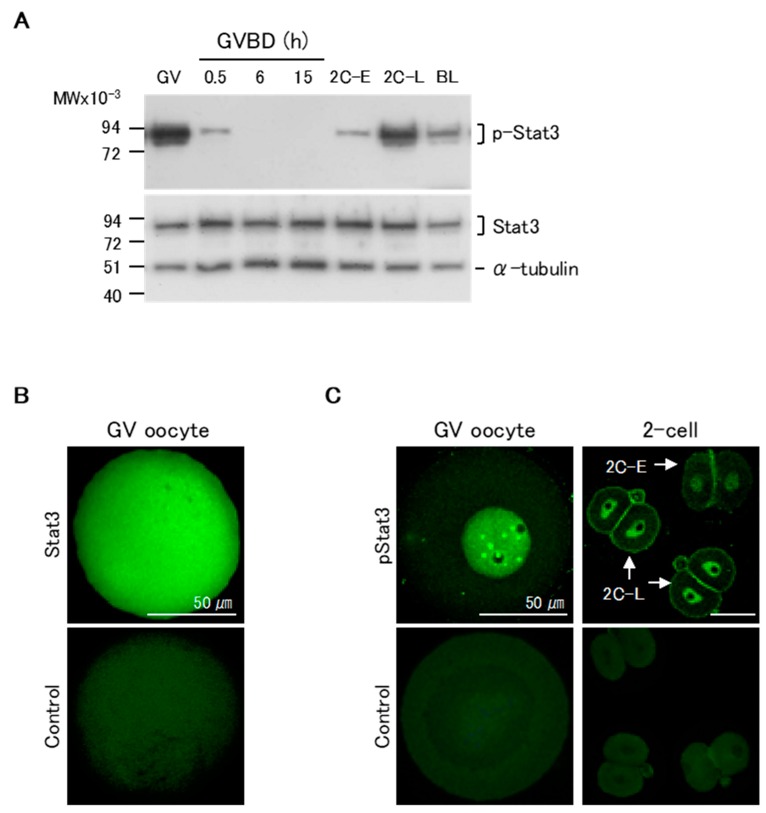
Patterns of expression of Stat3 and pStat3 in mouse oocytes and embryos. (**A**) Western blotting analysis. There is a considerable amount of pStat3 in the Germinal vesicle (GV) oocytes. At 0.5 h after germinal vesicle break down (GVBD), the amount of pStat3 decreases suddenly, and pStat3 cannot be detected until 15 h after GVBD. pStat3 is detected as a weak signal at the early 2-cell stage (2C-E) and a strong signal at the late 2-cell stage (2C-L). Conversely, a certain amount of Stat3 protein is detected at all stages. BL: blastocyst. (**B**) Immunocytochemical analysis reveals that the Stat3 protein is present in the whole cell. (**C**) Conversely, pStat3 exists in the nucleus in the GV oocyte and 2C-L (arrows). A weak signal of pStat3 is observed in the nucleus of 2C-E (arrow). Stat3 and pStat3 signals are shown in green color. As a negative control, the samples were incubated with the secondary antibody alone.

We first assessed the patterns of pStat3 expression in maturing oocytes and pre-implantation stage embryos by western blotting. pStat3 was highly expressed in GV oocytes (Figure 1A, upper panel). Following GVBD, pStat3 expression dramatically decreased at 0.5 h, and no signal was detected until 15 h of maturation, when oocytes were at the MII stage. In two-cell embryos, pStat3 expression was low at the early stage (2C-E) and high at the late stage (2C-L). pStat3 expression in GV oocytes and at 2C-L was higher than that in blastocysts, in which Stat3 is essential to maintain inner cell mass lineages [25]. Conversely, Stat3 protein expression was almost the same at all stages (Figure 1A, lower panel). We next examined Stat3 and pStat3 localization by immunocytochemical analysis. The non-phosphorylated Stat3 protein was ubiquitously expressed in oocytes (Figure 1B). Notably, a strong signal for pStat3 was detected in the nuclei of GV oocyte and 2C-L, but it was weak in the nucleus of 2C-E (Figure 1C); these results confirmed that the high pStat3 expression detected by western blotting reflects its localization in the nucleus at these stages.

### 2.2. pStat3 Localization

**Figure 2:**
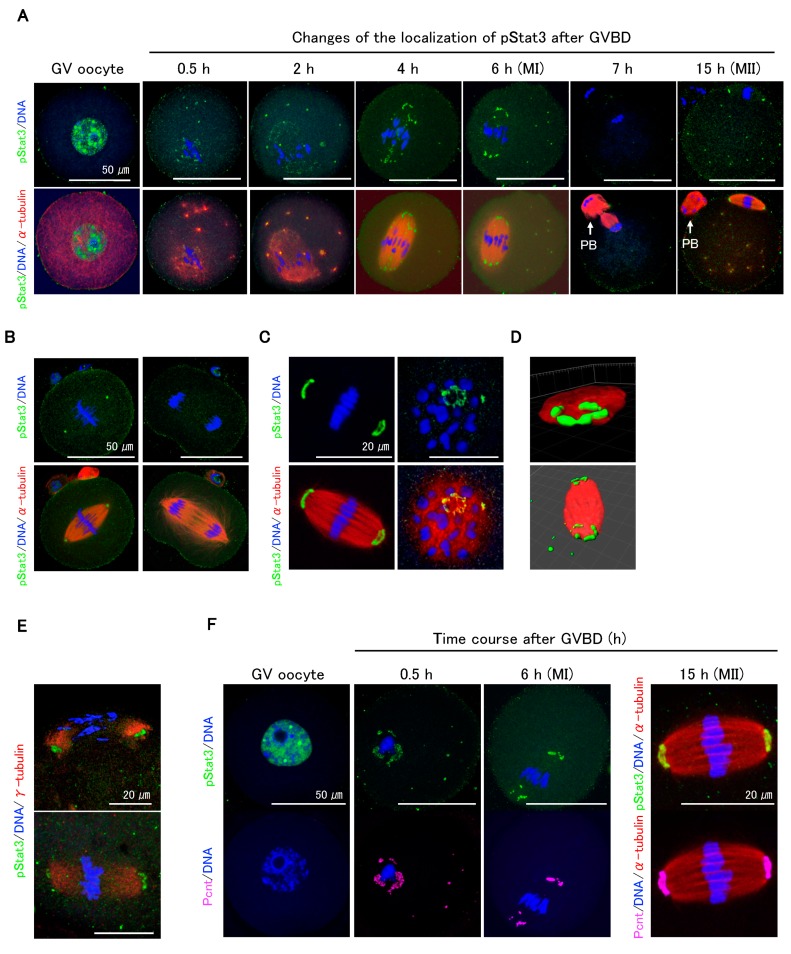
Dynamic changes of pStat3 localization during oocyte maturation. (**A**) Time course observation of pStat3 localization after germinal vesicle break down (GVBD). Immediately after GVBD, the accumulated pStat3 in the GV declines remarkably (0.5 h) and appears at microtubule asters (0.5 h, 2 h, and thereafter). The pStat3 emerges at the spindle (4 h) and microtubule-organizing centers (MTOCs) in the MI spindle (6 h), and it then disappears in the anaphase/telophase (7 h). The pStat3 localizes again at the MTOCs in the MII spindle (15h). Arrows indicate first polar body (PB). (**B**) The pStat3 localizes at the MTOCs in the M-phase (left figures), but not in the telophase (right figures) in the 1-cell stage. (**C**) The pStat3 exhibits a ring-shape at the MTOCs. The right-hand figures are views from the MTOC. (**D**) The ring-shape localization of pStat3 was also confirmed by a reconstructed 3D image with Imaris software 8.4. (**E**) pStat3 co-localizes with γ-tubulin at MTOCs in the MI (upper figure) and MII spindles (lower figure). The γ-tubulin exhibits a diffused pattern of expression. (**F**) Though pericentrin (Pcnt) is not detected in the GV, it emerges after GVBD and co-localizes with pStat3 during maturation. Different colors indicate pStat3 (green), α-tublin or γ-tublin (red), pericentrin (magenta), and DNA (blue).

Immunocytochemical analysis showed that pStat3 accumulated in GV oocytes (Figure 2A, GV oocyte) dramatically decreased following GVBD but remained in peri-chromosomes and appeared at the microtubule asters (Figure 2A, 0.5 and 2 h). As the oocytes proceeded to metaphase I (MI), pStat3 emerged at the meiotic spindle (Figure 2A, 4 h) and was arranged at MTOCs (Figure 2A, 6 h). pStat3 was not detected at anaphase/telophase (Figure 2A, 7 h). In MII spindle, pStat3 was relocalized at the polar MTOCs (Figure 2A, 15 h). We further investigated pStat3 localization pattern in one-cell embryo. At metaphase, pStat3 was localized at MTOCs (Figure 2B, left panels), consistent with its localization in MI and MII spindles (Figure 2A, 6 and 15 h). pStat3 was not detected at anaphase (Figure 2B, right panels), which is consistent with results in maturing oocytes at anaphase/telophase (Figure 2A, 7 h). pStat3 localized at MTOCs showed a ring-shaped pattern (Figure 2C), which was further confirmed by 3D reconstruction and surface rendering using Imaris (Figure 2D). Considering the pStat3 localization at MTOCs, double-staining immunocytochemistry with γ-tubulin or pericentrin was performed. Diffusely expressed γ-tubulin was co-localized with pStat3 at MTOCs in MI (Figure 2E, upper panel) and MII spindles (Figure 2E, lower panel). We examined pStat3 and pericentrin co-localization patterns in GV to MII oocytes. Pericentrin was not detected in the nucleus of the GV oocyte (Figure 2F). However, at 0.5 h following GVBD, pericentrin emerged around the chromosomes and microtubule asters (Figure 2F, 0.5 h) and was subsequently localized at MTOCs in MI (Figure 2F, 6 h) and MII spindles (Figure 2F, 15 h). Pericentrin expression pattern was consistent with pStat3 expression pattern in maturing oocytes. These findings suggest that γ-tubulin, pericentrin, and pStat3 are the components of MTOCs and are involved in meiotic spindle assembly during oocyte maturation.

### 2.3. Stat3/pStat3 Expression in Stat3^−/−^ Oocytes

**Figure 3:**
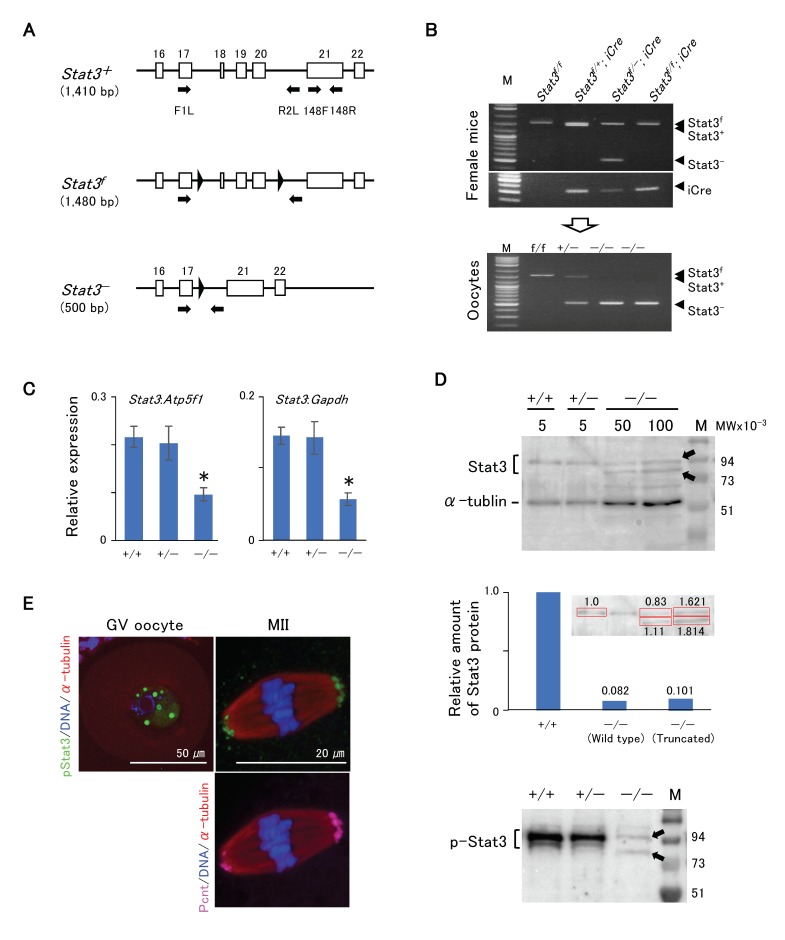
Phenotype of Stat3^−/−^ oocytes. (**A**) Schematic strategy of conditional knock out mouse [27] showing the positions of primers (arrows). Parentheses indicate the PCR product size for genotyping. (**B**) Genotyping PCR of oocytes with the primer set F1L/R2L. The *Stat3^−/−^* oocytes (lower panel) were obtained from *Stat3^f/f (f/−)^; Gdf9-iCre* female mice (upper panels). (**C**) RT-qPCR of oocytes with the primer set 148F/148R. In the *Stat3^−/−^* oocyte, the expression level of *stat3* is approximately 40% that of the *Stat3^+/+^* oocyte. * P < 0.05. (**D**) Western blotting of oocytes. Arrows indicate the wild type (88 kDa) and truncated (77 kDa) Stat3 and pStat3. Both proteins were detected only in the *Stat3^−/−^* oocytes. The middle panel shows quantification of the Western blotting signal for Stat3. The *Stat3^+/+^* oocyte (5 oocytes) was used as a reference. The amount of the Stat3 protein from 100 oocytes show approximately twice compared to that from 50 oocytes. Relative amounts of the Stat3 protein after calculation are shown, in which the total amount of Stat3 protein in the *Stat3^−/−^* oocyte is approximately 18% of the Stat3 protein in the *Stat3^+/+^* oocyte. Numbers (5, 50, and 100) show the oocyte numbers loaded per lane. (**E**) Immunocytochemical analysis shows proper localization of the pStat3 in the *Stat3^−/−^* oocyte. Note that the pStat3 signals (green) are weaker compared to those of the wild type shown in the Figure 2. Different colors indicate pStat3 (green), α-tublin (red), pericentrin (Pcnt) (magenta), and DNA (blue).

Figure 3A shows the schematic of development of *Stat3^flox/flox^* (*Stat3^f/f^*) or *Stat3^f^*^/^^−^ mice, with two *loxP* flank exons 18–20 (SH2 domain) of the *Stat3* allele exhibiting the conditional knockout of *Stat3* after crossing with a driver Cre Tg mouse [27]. We also successfully produced conditionally knocked out *Stat3^−/^^−^* oocytes from both *Stat3^f^*^/^^−^; *Gdf9-iCre* and *Stat3^f^*^/*f*^; *Gdf9-iCre* female mice (Figure 3B). We first conducted RT-qPCR analysis to ascertain the presence of *Stat3* mRNA in *Stat3^−/^^−^* oocytes. *Stat3* mRNA expression in *Stat3^−/^^−^* oocytes was relatively low, and it was ~40% in *Stat^+/^^+^* oocytes (Figure 3C). We performed western blotting to detect wild-type and/or truncated Stat3/pStat3 proteins in *Stat3^−/^^−^* oocytes. Stat3 protein was presented as two bands (Figure 3D, upper panel). The upper band was ~88 kDa, corresponding to the wild-type Stat3 in all genotype oocytes. The truncated Stat3 with 673 amino acid residues appeared as a band of ~77 kDa. Thus, the lower band detected only in *Stat3^−/^^−^* oocytes was most probably the truncated Stat3. Stat3 protein expression in *Stat3^−/^^−^* GV oocytes was respectively ~8.2% (wild-type Stat3) and ~10.1% (truncated stat3) relative to that in wild-type Stat3 protein in *Stat3^+/^^+^* GV oocytes (Figure 3D, middle panel). Moreover, two faint signals of pStat3 were detected in *Stat3^−/^^−^* oocytes (Figure 3D, lower panel), indicating phosphorylation of both Stat3 proteins. pStat3 was detected in the GV (Figure 3E, left panel) and concomitant with pericentrin expression at MTOCs in MII oocytes (Figure 3E, right panel), although the signal intensity was weaker than was that in wild-type oocytes (Figure 2F). As the anti-pStat3 antibody we used recognizes both wild-type and truncated pStat3, this finding may reflect the localization of both types of pStat3.

### 2.4. Effects of pStat3 Inhibition on Meiotic Spindle Assembly and Chromosome Segregation

**Figure 4:**
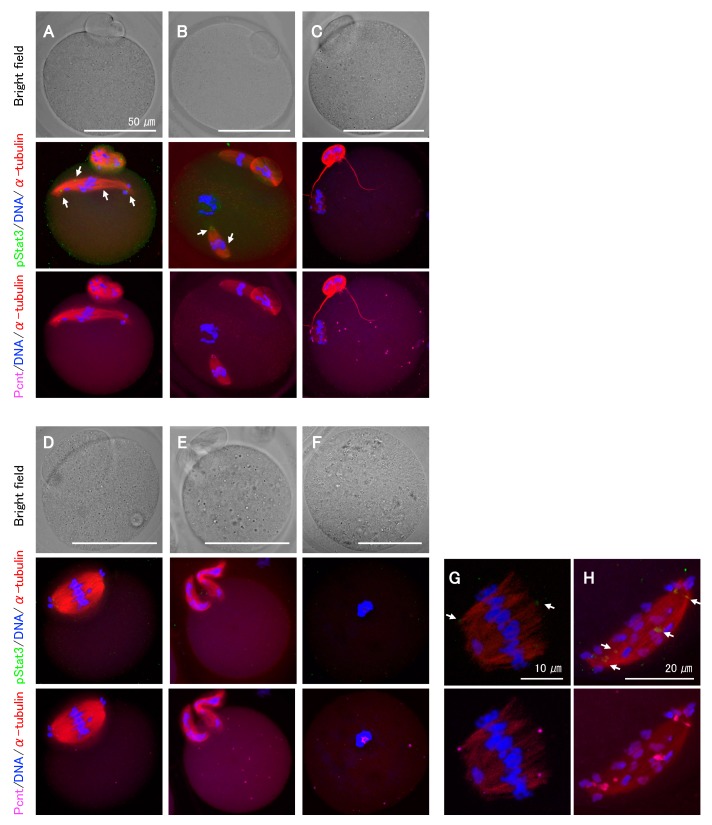
Representative features of abnormal MII oocytes treated with either Stat3 inhibitors or anti-pStat3 antibody. (**A**–**C**) These oocytes appear normal under bright field microscopy. (**A**) The spindle elongation and chromosomes aberration. (**B**) Two spindles involving chromosomes. A chromosome aggregation without spindle body is also seen. (**C**) Shortened and improper spindle formation. String-shaped microtubules are observed to extend from the polar body. (**D**) A larger polar body with expanded spindle assembly. (**E**) Two polar bodies with no spindle formation. (**F**) No polar body and spindle formation. Condensed chromosome with pericentrin is observed. (**G**,**H**) Higher magnification of abnormal spindles and chromosome mis-location. Note that the expression of pStat3 is either very faint and at an incorrect location (allows shown in **A**,**B**,**G**,**H**) or negative (**C**–**F**). Different colors indicate pStat3 (green), α-tublin (red), pericentrin (Pcnt) (magenta), and DNA (blue).

As the Stat3 protein in *Stat3^−/^^−^* oocytes is presumably functional, we next explored the effect of pStat3 inhibition by using small-molecule inhibitors, stattic and BP-1-102, and anti-pStat3 antibody. Stattic selectively inhibits Stat3 activation and dimerization via the reduction of Y705 phosphorylation [28]. Similarly, BP-1-102 blocks Y705 phosphorylation and dimerization in a dose-dependent manner [29]. We performed oocyte in vitro maturation (IVM) for 15 h in a culture medium supplemented with stattic or BP-1-102. In both cases, the frequency of abnormal MII oocytes increased in a dose-dependent manner (Table 1). Following stattic treatment, the first polar body (PB) emission was significantly decreased at 3 μM and entirely inhibited at 4 μM, confirming the identical sensitivity of *Stat3^+/^^+^* and *Stat3^−/^^−^* oocytes to stattic. Immunocytochemical analysis revealed abnormal phenotypes even in some apparently normal MII oocytes that emitted the first PB after treatments (Figure 4A–C). We classified the phenotypes into several patterns and observed chromosomal aberrations in all cases. For example, spindle microtubules were elongated rather than being arranged in a barrel configuration (Figure 4A,H); multiple spindle bodies were formed, each being involved chromosomes (Figure 4B); spindles were shortened and showed incorrect assembly (Figure 4C,G); a relatively larger PB containing an expanded spindle was formed (Figure 4D); or two PBs were formed without spindle assembly (Figure 4E). As shown in Figure 4F, at a higher concentration of inhibitors, i.e., 4 μM stattic (*Stat3^+/+^* and *Stat3^−/−^* oocytes) and 16 μM BP-1-102 (*Stat3^+/+^* oocytes), all oocytes exhibited a dark color (data not shown) and aggregated chromosomes without spindle formation. In all cases, pStat3 was absent or detected as a faint signal at an incorrect location concomitantly with pericentrin (Figure 4A,B,G,H). In some cases, only pericentrin was detected at the asters (Figure 4C,E) and in the aggregated chromosomes (Figure 4F). We further studied the effect of pStat3 inhibition via microinjection of anti-pY705 antibody. The results are shown in Table 2. Compared to PBS and IgG isotype control, the antibody significantly increased the frequency of abnormal MII oocytes. As shown in Figure 4, the phenotype was the same as that obtained with inhibitor treatments. Overall, these findings show that pStat3 is essential for meiotic spindle assembly and chromosome segregation.

### 2.5. Effects of pStat3 Disruption during IVM on Pre-Implantation Stage Embryos

The frequency of abnormal MII oocytes was dependent on inhibitor concentration. Such oocytes may show developmental arrest during the pre-implantation stages. To confirm the developmental ability of these oocytes, we performed in vitro fertilization (IVF) and in vitro embryo culture. The fertilization rate of two-cell embryos significantly decreased following stattic and BP-1-102 treatments in a dose-dependent manner (Table 3). Likewise, the rate of development to the blastocyst stage was significantly decreased. At the same dose of inhibitors, fertilization and development rates were lower than abnormal MII oocytes frequency. Oocytes treated with 3 μM stattic and 8 μM BP-1-102 could not develop beyond the two-cell stage likely because of the severe breakdown of spindle assembly and chromosome segregation in MII oocytes. We did not observe parthenogenesis. These findings demonstrate that the developmental ability of abnormal MII oocytes with ablated pStat3 function is significantly decreased during pre-implantation stages.

## 3. Discussion

Successful pregnancy is largely dependent on the quality of oocytes [30] in which proper spindle formation and chromosome segregation during maturation are pivotal. High-quality oocytes are essential for normal embryonic development after fertilization. In this study, we demonstrated the involvement of pStat3 in meiotic spindle assembly. Distinctly from somatic cells, mouse oocytes do not form conventional centriole-containing centrosomes; thus, meiotic spindle assembly occurs independently of centrosomes [31,32]. Spindle microtubules originate from MTOCs. Although the exact composition and structure of MTOCs remain uncertain, they express proteins such as γ-tublin and pericentrin in the form of classical pericentriolar materials [33,34,35,36]. The diffuse expression pattern of γ-tublin at MTOCs observed in this study was consistent with that observed in a previous study [37]. In addition, the ring-shaped expression pattern of pStat3 at the MTOCs was similar to that of pericentrin in mouse oocytes, as reported by Carabatosos et al. [33]. Therefore, pStat3 is probably a component of MTOCs. Pericentrin is involved in spindle formation. Oocytes showing low pericentrin expression show disrupted meiotic spindle assembly and organization with significant chromosomal aberrations [38]. Furthermore, embryonic fibroblasts of *pericentrin^−/^^−^* mouse exhibit spindle misorientation, which is associated with disruption of astral microtubules and loss of a unique set of centrosome proteins at spindle poles [39]. We also verified pStat3 localization at the centrosomes in several somatic cells, such as HeLa, COS-7, mouse embryonic fibroblasts, bovine embryonic fibroblasts, and porcine embryonic fibroblasts (Supplementary Figure S1). Thus, based on pStat3 and pericentrin co-localization at MOTCs and centrosomes, pStat3 likely interacts with pericentrin to regulate both meiotic and mitotic spindle formation.

Indeed, *Stat3^−/−^* oocytes matured to the MII stage without exhibiting an incorrect phenotype. Previous studies have used *Zp3-Cre* Tg mice for maternal *Stat3* knockout [25,26]. In the present study, we used *Gdf9-iCre* Tg mice, expecting complete maternal Stat3-null oocytes considering that the *Gdf9* promoter allows Cre expression in oocytes earlier at the follicular stage than does the *Zp3* promoter [24]. Nonetheless, we detected both *Stat3* mRNA and protein expression in *Stat3^−/−^* oocytes. The wild-type Stat3 protein is assumed to be translated from mRNA expressed prior to Cre expression. Whether the truncated pStat3 with the deletion of the SH2 domain is functional in the mouse oocytes remains unclear. According to Robker et al. [26], *Stat3^−/−^* oocytes derived from *Stat3^f/f^; Zp3-Cre* could mature normally. The *Stat3^f/f^* mice used were deletion mutants of exons 12–14, and they displayed frameshifted mRNA incapable of encoding functional proteins [40]. However, as we have mentioned, this may be because the maternal Stat3 expressed earlier than Cre driven by the *Zp3* promoter would remain functional in such oocytes. Discrepancies in the function of maternal Stat3 would be elucidated upon the creation of complete maternal Stat3-null oocytes.

Conditional targeting cannot completely disrupt maternal Stat3. Therefore, we used specific inhibitors of pStat3. Stattic [28] and BP-1-102 [29], which are nonpeptidic small-molecule inhibitors of Stat3, have been widely used in various studies, particularly in cancer research. Given the properties of these inhibitors, our aim was to inhibit Stat3 phosphorylation and inhibit its function in maturing oocytes. Moreover, we further applied the pStat3-specific antibody injection. Both *Stat3^+/^^+^* and *Stat3^−/^^−^* oocytes showed incorrect spindle assembly and chromosomal misarrangements, indicating that pStat3 regulates meiotic spindle assembly.

Recently, Morris et al. [10] demonstrated the contributions of Stat3 in the regulation of centrosome clustering in cancer cells via the stathmin/PLK1 pathway. Our findings are consistent with these results in terms of the novel function of Stat3/pStat3 in the regulation of spindle formation, extending to meiotic spindle assembly, which is independent of the centrosome. Precise mechanisms of action of Stat3/pStat3 in spindle microtubule formation will be revealed in the future.

## 4. Materials and Methods

### 4.1. Mice

ICR (Jcl:ICR, CLEA Japan, Inc., Japan), *Stat3^flox^* [27] (No. 016923, The Jackson Laboratory, Bar Harbor, ME, USA), and *Gdf9-iCre* Tg [24] (C57BL6/J; produced in-house) mice were housed at an ambient temperature of 23 °C ± 2 °C and 50% ± 10% humidity under a light/dark cycle (06:00-h day). Food (CE-2, CLEA, Tokyo, Japan) and water were provided *ad libitum*. *Stat3 ^f/f^* females were crossed with *Gdf9-iCre* Tg mice to generate *Stat3^f/^^+^; Gdf9-iCre* mice. These mice were back-crossed with *Stat3^f/f^* mice to generate *Stat3^f/^^f^; Gdf9-iCre* or *Stat3^f/^^−^; Gdf9-iCre* female mice, in which the oocytes are genetically *Stat3^−/^^−^*. Mice were maintained and handled in accordance with the guidelines issued by the NARO Institutional Animal Care and Use Committee (Approval No: H28-013, 1 April 2016) Day month year.

### 4.2. Genotyping

Tail snips of 18–20-day old mice were soaked in 90 μL of 50 mM NaOH, heated for 10 min at 98 °C, and neutralized with 10 μL of 1M Tris (pH 8.0). Primers used for *Stat3^flox^* mice were F1L (5′-aattggaacctgggaccaagtggccg-3′) and R2L (5′-agctggctcataggcaaaaacacctg-3′), and those used for *Gdf9-iCre* Tg mice were iCreF1 (5′-tggatgccacctctgatgaagtcag-3′) and iCreR1 (5′-tgattctcctcatcaccagggacac-3′). PCR was performed for 30 cycles at 98 °C for 10 s and 68 °C for 90 s using the EmeraldAmp PCR Master Mix (TaKaRa Bio Inc., Shiga, Japan). For oocyte genotyping, two GV oocytes, after removal of the zona pellucida by acidic Tyrode’s solution (T1788, Sigma-Aldrich, St. Louis, MO, USA), were introduced into a PCR tube containing 1.5 μL 50 mM NaOH and heated for 5 min at 95 °C After adding 6 μL of 30 mM Tris (pH 8.0), PCR was conducted for 35 cycles, as described above.

### 4.3. Collection of GV Oocytes and IVM

We injected 8–12-week old female mice with 5 IU eCG (Serotropin; ASKA Animal Health Co., Ltd., Tokyo, Japan) to stimulate preovulatory follicle development and mechanically collected cumulus-enclosed oocyte complexes after 46 h in TYH medium [41] supplemented with 20 mM HEPES (TYH-HEPES) and 50 μM 3-isobutyl-1-methylxanthine (IBMX; I5879, Sigma-Aldrich). After the GV oocytes were freed from the cumulus cells using a fine glass capillary with a mouthpiece, they were transferred to TYH medium supplemented with 5% (v/v) FCS (SH30070.03, HyClone, Thermo Fisher Scientific, Waltham, MA, USA) (TYH-FCS) overlaid with paraffin liquid (#26137-85, nacalai tesque, Inc., Kyoto, Japan) and cultured in a humidified atmosphere of 5% CO_2_ in air at 38 °C. Oocytes that completed GVBD within 60 min in TYH-FCS were selected, and IVM was performed for 15 h. For western blotting and immunocytochemical analyses, oocytes at the indicated time points after GVBD were collected.

### 4.4. IVF

After IVM, oocytes were washed with modified HTF (mHTF) [42] and transferred to a fertilization medium containing mHTF and supplemented with 0.25 mM reduced glutathione (GSH, G4251, Sigma-Aldrich) [43]. Sperm were collected from the cauda epididymidis of matured ICR mice and capacitated in 200 µl TYH drop covered with paraffin liquid for 1 h in a humidified atmosphere of 5% CO_2_ in air at 38 °C. Sperm were added to 100 µl of fertilization medium at a final concentration of 3 × 10^2^ sperms/µl. After insemination for 2 h, embryos were washed and cultured in 50 μL KSOM [44] in a humidified atmosphere of 5% CO_2_ in air at 38 °C. The rate of development of embryos was recorded every 24 h until 96 h had elapsed.

### 4.5. Evaluation of Small-Molecule Stat3 Inhibitors

Two small-molecule inhibitors of Stat3 activation—stattic (#2798, Tocris Bioscience, Bristol, UK) and BP-1-102 (#573132, Calbiochem, Darmstadt, Germany)—were used. As stock solutions, 50 mM stattic and 80 mM BP-1-102 were prepared with DMSO (D2650, Sigma-Aldrich) and kept at −20 °C. GVBD oocytes were transferred to TYH-FCS containing 1, 2, 3, and 4 μM stattic or 2, 4, 8, and 16 μM BP-1-102, and IVM was performed. As a control (0 μM), we added 0.008% DMSO, which is equivalent to a DMSO concentration of 4 μM stattic in TYH-FCS. After 15 h of IVM, oocytes were subjected to either immunocytochemistry or IVF.

### 4.6. Evaluation of Anti-pStat3 Antibody Injection

Anti-phospho Stat3 (Tyr705) rabbit mAb (#9145, Cell Signaling Technology, Danvers, MA, USA) and isotype control rabbit mAb IgG (#3900, CST) were purified and concentrated with PBS using Amicon Ultra-0.5 (50 kDa, Merck). We subjected small aliquots of purified antibody and IgG to polyacrylamide gel electrophoresis, stained them using Rapid Stain CBB Kit (Nacalai Tesque), and determined the concentration as being 0.4 μg/μL. Purified anti-pStat3 antibody, control IgG, and PBS were microinjected into oocytes within 1 h after GVBD in TYH-HEPES, and the oocytes were then cultured in TYH-FCS.

### 4.7. Western Blotting

Western blotting was performed according to standard procedures. Proteins were extracted by using a double-strength SDS sample buffer, heated for 5 min at 90 °C, and stored at −80 °C until use. Unless otherwise stated, 50 oocytes or embryos per lane were used. After blocking with 5% skim milk (BD Biosciences) in TBST (0.1% Tween 20) for 1 h, the membranes were washed with TBST and probed with anti-phospho Stat3 (Tyr705) (1:1000, #9145, CST), anti-Stat3 (1:1000, #12640, CST), or anti-α-Tublin antibody (1:2000, #2125, CST) with 5% Blocking One-P (Nacalai Tesque) in TBST at 4 °C for overnight. The membranes were washed with TBST and incubated with HRP-conjugated anti-rabbit IgG secondary antibody (1:2000, #7074, CST) in blocking buffer at room temperature for 1 h. The membranes were washed with TBST and processed using the Chemi-Lumi One Ultra (Nacalai Tesque). Immunoblots were visualized using either Hyperfilm ECL (GE Healthcare) or ImageQuant LAS 500 (GE Healthcare). Quantification was performed using ImageQuant TL8.1 (GE Healthcare, Chicago, IL, USA).

### 4.8. Immunocytochemistry

Samples were fixed in 4% paraformaldehyde in PBS for 15 min at room temperature, washed with PBS, and treated with 0.3% Triton X-100 and 2% BSA in PBS (PBS-XB) for 1 h. The samples were incubated overnight at 4 °C with primary antibody against phospho Tyr 705 Stat3 (1:200, GTX118000, GeneTex or 1:100, #9145, CST) or Stat3 (1:500, #12640, CST) with 0.3% Triton X-100 in PBS (PBS-X). The samples were washed with PBS-X and then incubated with Alexa Fluor 488-anti-rabbit secondary antibody (1:500, A-11070, TFS) in PBS-XB at room temperature for 1 h. After washing with PBS-X, the samples were incubated anti-α-Tublin-Alexa Fluor 594 antibody (1:200, M175-A59, MBL, Nagoya, Japan) in PBS-XB at room temperature for 1 h. As a negative control, the samples were incubated with the secondary antibody alone. For double staining, anti-mouse pericentrin (1:400, #611815, BD Biosciences, San Jose, CA, USA) and Alexa Fluor 647-anti-mouse antibodies (1:500, A-21235, TFS), or anti-mouse-γ-tublin (1:100, sc-17787, Santa Cruz Biotechnology, Dallas, TX, USA) and Alexa Fluor 594-anti-mouse antibodies (1:500, A-1105, TFS) were added to the reaction described above as primary and secondary antibodies, respectively. The oocytes were transferred onto a slide glass with SlowFade Diamond Antifade Mountant in DAPI (S36968, TFS). To avoid squashing the samples, the vaseline–paraffin mixture (9:1) was spotted between the slide glass and the coverslip in advance. The specimens were imaged using a Zeiss LSM780 confocal microscope.

### 4.9. RT-qPCR

RNA was extracted from 20 GV oocytes using the PicoPure RNA Isolation Kit (TFS). After treatment with DNaseI, first-strand cDNA was synthesized with oligo dT primers using the PrimeScript II 1st strand cDNA Synthesis Kit (TaKaRa Bio), following the manufacturer’s instructions. RT-qPCR was performed using a LightCycler 480 SYBR Green I (Roche) on a LightCycler 480 (Roche). The primers used for internal control were *Atp5f1* and *Gapdh*, which are components of the Mouse Housekeeping Gene Primer Set (TaKaRa Bio). Primer used for *Stat3* were 148F (5′-accaacatcctggtgtctccacttg-3′) and 148R (5′-agatgaacttggtcttcaggtacggg-3′).

### 4.10. Statistics

All data were obtained from five (*Stat3^+/^^+^* oocytes) or three (*Stat3^−/^^−^* oocytes) independent experiments and analyzed by one-way ANOVA followed by Tukey’s test, using general linear models in Statistical Analysis System (SAS Institute Inc., Cary, NC, USA). Percentage data were arcsine-transformed before analysis. Data were expressed as mean ± standard error of the mean. P values less than 0.05 were considered to be statistically significant.

## Figures and Tables

**Table 1:** Effects of the pStat3 inhibitors, Stattic, and BP-1-102, on in vitro maturation.

Inhibitors	No. of Oocytes Examined	No. (%) of Oocytes Extruded 1st PB	No. (%) of Abnormal MII Oocyte*
(*Stat3^+/+^*)			
Stattic			
0 μM**	102	99 (97.2 ± 1.8)^a^	3 (2.8 ± 1.8)^a^
1 μM	102	100 (98.0 ± 1.2)^a^	10 (9.9 ± 2.8)^a^
2 μM	102	98 (96.2 ± 1.7)^a^	45 (44.3 ± 6.3)^b^
3 μM	102	25 (24.8 ± 11.0)^b^	93 (86.3 ± 4.3)^c^
4 μM	102	0 (0)^c^	102 (100)^c^
BP-1-102			
0 μM**	100	99 (99.0 ± 1.0)^a^	3 (3.0 ± 2.0)^a^
2 μM	100	100 (100)^a^	5 (5 ± 1.6)^a^
4 μM	100	98 (96.2 ± 1.2)^a^	13 (13 ± 3.4^a^
8 μM	100	96 (96.0 ± 1.9)^a^	62 (62 ± 6.8)^b^
16 μM	100	82 (82.0 ± 5.1)^b^	100 (100)^c^
(*Stat^−/−^*)			
Stattic			
0 μM**	64	62 (95.6 ± 4.4) ^a^	4 (6.5 ± 0.4) ^a^
1 μM	59	58 (98.0 ± 2.0) ^a^	7 (12.5 ± 1.5) ^a^
2 μM	62	56 (91.8 ± 2.0) ^a^	28 (45.8 ± 7.2) ^b^
3 μM	60	13 (21.9 ±3.8) ^b^	53 (88.6 ± 4.0) ^c^
4 μM	50	0 (0) ^c^	50 (100) ^d^

* Representative abnormal phenotypes are shown in Figure 4. ** As a control, 0.008% DMSO was included in the medium. Percentages are shown ± SEM. Different superscripted letters within the inhibitor denote significant differences in five (*Stat3^+/+^*) and three experiments (*Stat^−/−^*) (*P* < 0.05).

**Table 2:** Effects of anti-pStat3 antibody injection on in vitro maturation.

Microinjection	No. of Oocytes Examined	No. (%) of Oocytes Extruded 1st PB	No. (%) of Abnormal MII Oocyte*
Anti-pStat3 antibody	270	239 (87.8 ± 2.1)^a^	72 (27.5 ± 2.5)^a^
Isotype control IgG	245	237 (97.0 ± 1.2)^ab^	9 (3.5 ±1.3)^b^
PBS	213	208 (98.6 ± 1.1)^b^	8 (4.4 ± 1.9)^b^

* Representative abnormal phenotypes are shown in Figure 4. Percentages are shown ± SEM. Different superscripted letters denote significant differences in five experiments (*P* < 0.05).

**Table 3:** Effects of pStat3 inhibitors on embryo development after in vitro fertilization.

Inhibitors	No. of Oocytes Examined	No. (%) of Oocytes Extruded 1st PB	No. (%) of Embryos Developed to			
			2-Cell	4-Cell	8-Cell/Morula	Blastocyst
Stattic						
0 μM *	184	183 (99.4 ± 0.6) ^a^	164 (89.0 ± 3.3) ^a^	129 (69.9 ± 2.8) ^a^	123 (66.5 ± 3.8) ^a^	113 (61.2 ± 2.5) ^a^
1 μM	188	186 (90.0 ± 0.7) ^a^	113 (60.0 ± 5.2) ^b^	35 (18.4 ± 1.9) ^b^	34 (17.9 ± 2.1) ^b^	31 (16.4 ± 2.8) ^b^
2 μM	194	190 (97.9 ± 1.4) ^a^	114 (57.4 ± 5.3) ^b^	20 (9.7 ± 3.0) ^c^	18 (8.6 ± 2.7) ^c^	111 (5.2 ± 1.8) ^c^
3 μM	199	70 (34.8 ± 9.8) ^b^	37 (18.4 ± 7.4) ^c^	0 (0) ^d^	0 (0) ^d^	0 (0) ^d^
BP-1-102						
0 μM *	95	95 (100)	83 (87.4 ± 4.7) ^a^	68 (71.9±3.3) ^a^	68 (71.9±3.3) ^a^	62 (65.3 ± 3.3) ^a^
2 μM	105	104 (98.9 ± 1.1)	88 (83.6 ± 8.2) ^ab^	52 (49.3±5.4) ^b^	48 (44.9±3.8) ^b^	46 (42.7 ± 3.4) ^b^
4 μM	93	92 (99.2 ± 0.8)	61 (66.6 ± 3.4) ^b^	24 (26.8±3.4) ^b^	24 (26.8±3.4) ^b^	20 (22.1 ± 1.8) ^c^
8 μM	99	95 (96.0 ± 2.5)	21 (21.4 ± 0.8) ^c^	0 (0) ^c^	0 (0) ^c^	0 (0) ^d^
Non-IVF **	80	79 (99.6 ± 0.4)	0 (0)	0 (0)	0 (0)	0 (0)

* As a control, 0.008% DMSO was included in the maturation medium. ** MII oocytes matured without inhibitors were cultured without IVF. Percentages are shown ± SEM. Different superscripted letters within the inhibitor denote significant differences in five experiments (*P* < 0.05).

## Supplementary Materials

The following are available online at https://www.mdpi.com/1422-0067/21/4/1220/s1.
