# Supplementary material for: Dynamic Changes in pStat3 Are Involved in Meiotic Spindle Assembly in Mouse Oocytes
Source: Int J Mol Sci. 2020 Feb 12;21(4):1220. doi: 10.3390/ijms21041220 (PMC7072877; doi:10.3390/ijms21041220)
Supplement: Supplementary file 1 [file ijms-21-01220-s001.pdf]

Figure S1: pStat3 localizes at the centrosome in various mammalian somatic cells.

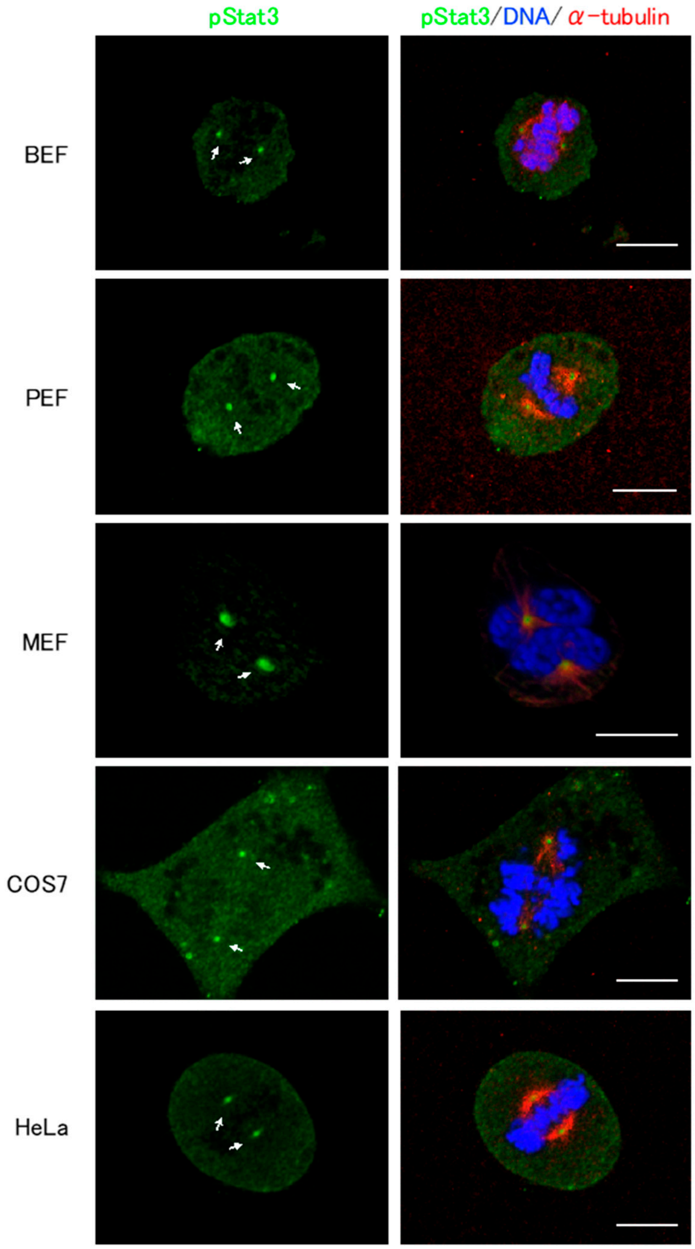

Figure S1. pStat3 localizes at the centrosome (arrows) in various mammalian somatic cells. BEF: primary bovine embryonic fibroblasts. PEF: primary porcine embryonic fibroblasts. MEF: primary mouse embryonic fibroblasts. Scale bars: 10 μm.
